# Forced Degradation Studies to Identify Critical Process Parameters for the Purification of Infectious Measles Virus

**DOI:** 10.3390/v11080725

**Published:** 2019-08-07

**Authors:** Daniel Loewe, Julian Häussler, Tanja A. Grein, Hauke Dieken, Tobias Weidner, Denise Salzig, Peter Czermak

**Affiliations:** 1 Institute of Bioprocess Engineering and Pharmaceutical Technology, University of Applied Sciences Mittelhessen, Wiesenstraße 14, 35390 Giessen, Germany; 2 Faculty of Biology and Chemistry, University of Giessen, Heinrich-Buff-Ring 17, 35392 Giessen, Germany; 3 Fraunhofer Institute for Molecular Biology and Applied Ecology (IME), Project group Bioresources, Winchesterstr. 3, 35394 Giessen, Germany

**Keywords:** temperature, pH, buffer, ionic strength, osmolality, isoelectric point, shear stress, Measles virus, stability

## Abstract

Oncolytic measles virus (MV) is a promising treatment for cancer but titers of up to 10^11^ infectious particles per dose are needed for therapeutic efficacy, which requires an efficient, robust, and scalable production process. MV is highly sensitive to process conditions, and a substantial fraction of the virus is lost during current purification processes. We therefore conducted forced degradation studies under thermal, pH, chemical, and mechanical stress to determine critical process parameters. We found that MV remained stable following up to five freeze–thaw cycles, but was inactivated during short-term incubation (< 2 h) at temperatures exceeding 35 °C. The infectivity of MV declined at pH < 7, but was not influenced by different buffer systems or the ionic strength/osmolality, except high concentrations of CaCl_2_ and MgSO_4_. We observed low shear sensitivity (dependent on the flow rate) caused by the use of a peristaltic pump. For tangential flow filtration, the highest recovery of MV was at a shear rate of ~5700 s^−1^. Our results confirm that the application of forced degradation studies is important to identify critical process parameters for MV purification. This will be helpful during the early stages of process development, ensuring the recovery of high titers of active MV particles after purification.

## 1. Introduction

Cancer remains one of the most common diseases, with 17 million new cases reported in 2018 and 9.6 million deaths [1]. The high mortality rate reflects the resistance of late-stage cancer to traditional therapy, such as radiotherapy, surgery, and chemotherapy. Several new strategies have been developed to improve the prognosis for late-stage cancer patients, including oncolytic viruses that target cancer cells and kill them. Drugs based on oncolytic Adenovirus, Herpes simplex virus, or Echovirus have been approved in China, the USA, the EU, or Latvia [2], and many others are undergoing clinical trials [3,4]. One promising candidate is measles virus (MV), because of its natural affinity for cancer cells [5] and the excellent safety profile of measles vaccines. Clinical trials involving oncolytic MV have confirmed its potency against late-stage cancers [6], but massive titers are required for therapeutic efficacy, ranging from 10^8^ to 10^11^ infectious virus particles per dose [6,7]. Current production processes for measles vaccines do not yield sufficient MV titers for cancer treatment, and new processes are therefore needed. For oncolytic MV, only one production strategy was published by the Mayo Clinic without mentioning any recovery and impurity depletion [8]. They produce the MV in cell factories and purify it with filtration-based methods, e.g., depth filtration and tangential flow filtration (TFF). Newer studies have improved the upstream processing [9,10] by changing the static production process to a dynamic stirred tank reactor (STR) process. This, together with an optimal host cell line [11] and the implementation of online monitoring, has resulted in MV titers of up to 10^10^ infectious particles per mL [12,13], while the standard static cell culture process leads to a titer of 10^8^ infectious particles per mL [11]. The production bottleneck has therefore shifted to downstream processing (DSP), because many infectious MV particles are currently lost during purification. MV is an enveloped virus, and the fragile lipid envelope is sensitive to chemical and physical extremes [14,15,16,17]. It is therefore necessary to investigate the effect of physicochemical parameters on the stability of MV during DSP/formulation to ensure the recovery of high titers of active MV particles.

To our knowledge, only we and a research group from Croatia investigate DSP unit operations for oncolytic MV [18]. Our DSP strategy combined several filtration and chromatography unit operations to achieve clarification, purification, and isolation. During these unit operations, the MV was exposed to many stress factors including freeze–thaw stress; thermal stress; mechanical stress (shear stress, e.g., caused by pumping and filtration); chemical stress due to the presence of additives; and the stress induced by changing the pH, ionic strength, and osmolality of the feed stream. The impact of stress parameters can be investigated using forced degradation (FD) studies, which are recommended in several ICH (International Conference of Harmonisation) guidelines for the pharmaceutical industry [19]. In particular, ICH guidelines Q8(R2) and Q11 highlight the importance of FD studies in pharmaceutical development generally as well as in the development and manufacturing of drug substances [20,21]. FD studies are already used to evaluate the process-related stability of protein-based therapeutics and vaccines under certain stress factors [19,22]. However, we are unaware of any published FD study focusing on the stress-related response of oncolytic MV or other oncolytic viruses. We are convinced that FD studies can significantly improve the recovery of active MV particles during DSP by indicating critical parameters and boundary values, thus accelerating process development.

We therefore conducted FD studies to determine critical parameters that reduce the infectivity of MV and therefore limit the yield of infectious particles during purification. We focused on the DSP-relevant parameters of repetitive freeze–thawing, temperature, composition of the feed (serum-containing and serum-free medium), osmolality, ionic strength, buffers, inorganic salts, additives, Ph, and shear stress. We also determined the isoelectric point (pI) of MV (the point at which the net charge is zero), allowing us to avoid aggregation and to adjust the surface charge in order to optimize DSP steps that rely on electrostatic interactions. The FD studies are a valuable starting point to develop a purification strategy and build the basis of an efficient downstream process.

## 2. Materials and Methods

### 2.1. Virus Production and Analysis of Infectivity

The MV strain MVvac2 GFP (P) was propagated in Vero cells (#CCL-81, ATCC), as previously described [12]. MVvac2 GFP (P) is an infectious recombinant vaccine strain, kindly provided by Dr. Michael Muehlebach (Paul-Ehrlich-Institute, Langen, Germany). MV suspensions were produced by dynamic fermentation in a stirred-tank reactor [12]. Briefly, the Vero cells were grown on Cytodex 1 carriers (3 g L^−1^) (GE Healthcare, Little Chalfont, UK) either in serum-containing medium (DMEM-HG supplemented with 10% (*v/v*) fetal calf serum, both from Biochrom, Cambridge, UK, and 10 mM HEPES) or serum-free medium (VP-SFM, ThermoFisher Scientific, Waltham, MA, USA) [12]. At the end of fermentation, the MV-containing supernatant was harvested by passing through a 5 µm Polygard CR Opticap XL-Capsule (Merck Millipore, Darmstadt, Germany) to remove carriers, cells, and cell debris. The supernatant was then centrifuged for 10 min at 300 *g* in a Heraeus Multifuge X1R (Thermo Fisher Scientific, Waltham, MA, USA). The MV titer was established by measuring the infectivity of the virus using the 50% tissue culture infective dose (TCID_50_) method [11] and converting this to a titer, as previously described [23,24].

### 2.2. Influence of Freeze–Thaw Cycles on MV Infectivity

The clarified MV suspension was divided into 1-mL aliquots in 1.5-mL tubes, which were frozen at –80 °C for at least 2 h. The tubes were then thawed by incubating for 15 min in a water bath at room temperature. This procedure was repeated for 1, 3, 5, and 10 cycles, with some aliquots removed for testing after cycles 1, 3, 5, and 10. The experiments were conducted in triplicates. The infectivity of the virus was determined as above, with the untreated MV suspension as a positive control.

### 2.3. Influence of Heat on MV Infectivity

We incubated 1-mL aliquots of the clarified MV suspension for 1 or 2 h at 15, 27, 35, 45, 50, 55, and 65 °C using either a WNB 22 water bath (Memmert, Germany) or a Thermomixer compact heating plate (Eppendorf, Hamburg-Eppendorf, Germany). Following the incubation period, the aliquots were immediately frozen at –80 °C to stop any further deterioration. The experiments were conducted in triplicates. The infectivity of the virus was determined as above, with the untreated MV suspension as a positive control.

### 2.4. Influence of pH on MV Infectivity and Aggregation

The clarified MV suspension was diluted 1:10 with 20 mM Tris-HCl (pH 7.5–9.0) or 20 mM citrate-phosphate buffer (pH 3.0–7.0) in steps of 0.5 along the pH scale for the used buffers. Experiments were conducted at room temperature and in triplicates. The infectivity of the virus was determined as above immediately after mixing the virus suspension with the buffer or after incubation for 1 h at room temperature followed by freezing at –80 °C. A suspension of MV diluted 1:10 in 20 mM Tris-HCl (pH 7.4) was used as a positive control.

The size distribution of the MV particles was investigated by dynamic light scattering (DLS) using a Zetasizer Nano ZS90 device (Malvern Instruments, Malvern, UK) and a low-volume cuvette (Sarstedt, Sarstedt, Germany). The suspension was stored on ice and mixed with buffer immediately before the initial measurement, and the measurement was repeated after incubation for 1 h at room temperature.

### 2.5. Influence of Osmolality and Ionic Strength on MV Infectivity

The clarified MV suspension (441 ± 48 mOsm kg^−1^; 16.8 ± 1.5 mS cm^−1^) was diluted 1:10 with sucrose or glucose solutions for omsolality measurements, and with sodium chloride or potassium chloride solutions for ionic strength measurements prepared in Milli-Q water (Merck, Darmstadt, Germany). The adjusted ranges were ~40–1100 mOsm kg^−1^ and ~3–62 mS cm^−1^. Aliquots were incubated for 24 h at 4 °C before freezing at –80 °C. The ionic strength was measured indirectly by the conductivity, using a SevenGo Duo Pro device (Mettler Toledo, Zurich, Switzerland). Osmolality was determined by using a Type 6 osmometer (Löser, Berlin, Germany). The experiments were conducted in triplicates. The infectivity of the virus was determined as above with an MV suspension, diluted 1:10 in PBS as a positive control.

### 2.6. Influence of Buffers and Additives on MV Infectivity

The following buffers were tested at two concentrations (20 mM and 100 mM prepared in Milli-Q water): Tris, HEPES, phosphate buffer (Na_2_HPO_4_/NaH_2_PO_4_), and citrate-phosphate buffer. In each case, the pH was set to 7.4 to avoid pH-dependent effects. We also tested the following additives, all of which were prepared in 100 mM Tris-HCl (pH 7.4): NaCl (up to 2 M), MgSO_4_ and CaCl_2_ (both up to 1.5 M), and L-arginine (up to 0.2 M). For all buffers and additives, clarified MV suspensions were diluted 1:10 in 1.5-mL tubes and were incubated for 24 h at 4 °C before freezing at −80 °C. The experiments were conducted in triplicates. The infectivity of the virus was determined as above with an MV suspension diluted 1:10 in PBS as a positive control.

### 2.7. Influences of Shear Stress on MV Infectivity

The effect of shear stress on MV was evaluated using the Sartoflow Slice 200 Benchtop System (Sartorius, Göttingen, Germany) fitted with a Tandem 1082 pump head and Phar Med #15 tubing, with and without a Sartorius 100-nm polyether sulfone (PES) membrane (filtration area 0.02 m²). For each run, 50 mL of the clarified virus suspension was loaded in the feed reservoir and pumped at different flow rates (0, 50, 100, 150, 200 and 250 mL min^−1^) for a maximum of 2 h. We took 1-mL samples after 1 and 2 h, which were frozen at −80 °C, and the infectivity of the virus was determined as above, with the untreated suspension as a positive control. When the membrane was fitted, the experiments were carried out in independent triplicates with closed permeate valve and the membrane was cleaned with 1 M NaOH.

### 2.8. Determination of the Shear Rate and Shear Stress

Shear stress can be calculated using the viscosity of a fluid and the shear rate, as shown in Equation (1)

(1) $$\tau =\mu \cdot \dot{\gamma }$$

where ***τ*** is the shear stress (mPa), ***μ*** is the dynamic viscosity (N m^−2^ s) and $\dot{\gamma }$ is the shear rate (s^−1^). The shear rate is dependent on the geometry of the flow channel. For a tube with a circular flow area, the shear rate is dependent on the volumetric flow rate ***Q*** (m^3^ s^−1^) and the tube inner radius $R_{id}$ (m), as shown in Equation (2):

(2) $$\dot{\gamma }=\frac{4\cdot Q}{\pi \cdot {R_{id}}^{3}}$$

Based on the shear rate, the resulting shear stress can be calculated using Equation (3)

(3) $$\tau_{av}=\frac{4\cdot \mu \cdot Q}{\pi \cdot {R_{ir}}^{3}}$$

For a rectangular geometry such as the flow channel of a flat sheet membrane, the shear rate can be calculated using Equation (4)

(4) $$\dot{\gamma }=\frac{6\cdot Q}{B\cdot H^{2}}$$

where ***B*** is the width of the flow channel (m) and ***H*** is the thickness of the flow channel (m). The shear stress can then be determined using Equation (5)

(5) $$\tau_{av}=\frac{6\cdot \mu \cdot Q}{B\cdot H^{2}}$$

### 2.9. Statistical Analysis

Each experiment was carried out three times (*n* = 3, three replicates). The three replicates were used to calculate the mean and standard deviation. To compare the mean values, a t-test (two sample test with known variances) was performed.

## 3. Results

### 3.1. Freeze–Thaw Stress

**Figure 1:**
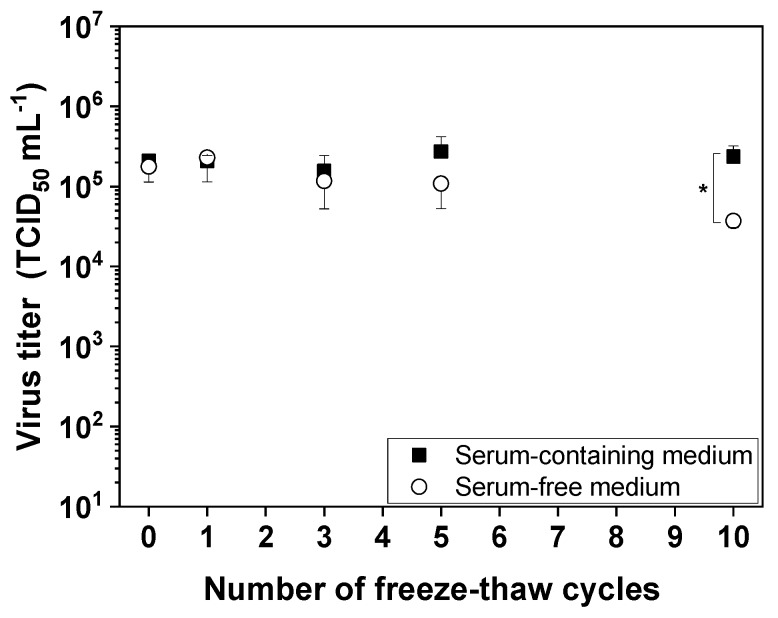
Influence of repeated freeze–thaw cycles on measles virus (MV) infectivity. MV aliquots were frozen at –80 °C for at least 2 h and thawed for 15 min in a water bath at room temperature, representing one cycle. Up to 10 freeze–thaw cycles were performed. Data are means plus standard deviations (*n* = 3; * *p* < 0.05).

A high freeze–thaw stability of the MV would allow an increased manufacturing flexibility. If the MV can be frozen at −80 °C without losing infectivity, the storage of MV suspension between two DSP unit operations is easily possible. Further, the control samples for QC analysis can be stored frozen if it is validated that the storage does not influence the quality and quantity of the MV. It is therefore necessary to determine the influence of freeze–thaw cycles on MV infectivity. Accordingly, we subjected cell-free MV aliquots stored in either serum-containing or serum-free medium to up to 10 freeze–thaw cycles to investigate the impact on MV infectivity and the influence of serum, if any. We found that repetitive freezing and thawing had no effect on the infectivity of MV in serum-containing medium, whereas 10 cycles reduced the infectivity of MV in serum-free medium significantly (*p* < 0.05) by approximately one order of magnitude (Figure 1). Our results therefore showed that serum protects MV from damage caused by freeze–thaw cycles, but that up to five cycles have a negligible effect on infectivity even if the virus is stored in serum-free medium.

### 3.2. Thermal Stress

**Figure 2:**
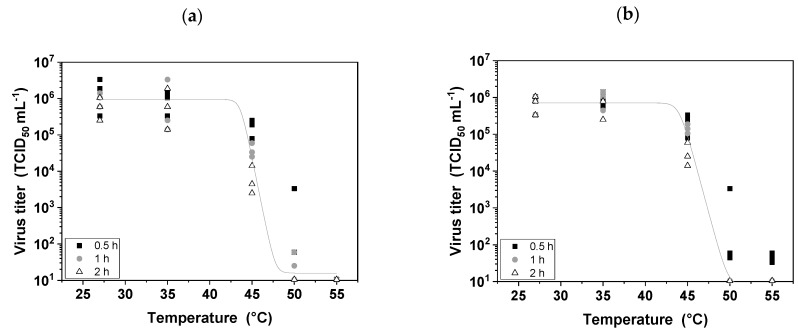
Short-term thermostability of MV. Suspensions of MV in serum-containing medium (**a**) or serum-free medium (**b**) were incubated for 30 min, 1 h, and 2 h at temperatures between 27 and 60 °C. Experiments were conducted in triplicate, and all data points are shown.

The thermosensitivity of MV has been addressed during upstream production by setting a fermentation temperature that balances host cell activity with the retention of MV infectivity [9]. In contrast, the thermosensitivity of MV is less problematic during DSP because the temperature can be maintained at 4 °C and residence times are low, especially in laboratory-scale processes. Nevertheless, DSP is more economical at room temperature so it is important to determine the stability of the virus over a range of temperatures and residence times. We investigated a broad temperature range (15–65 °C), because we aim to gain a deeper understanding of short-term MV thermal stability. We found that MV remained stable at temperatures below 35 °C for short residence times (30 min, 1 h, and 2 h), which are commensurate with typical DSP timescales. However, at higher temperatures the stability of the virus declined with longer residence times, and was similar whether or not serum was present in the medium, indicating that serum has no protective effect (Figure 2). Incubating the MV suspension at 45 °C for 2 h reduced the infectivity by approximately two orders of magnitude (*p* < 0.05), whereas incubation at 55 °C completely inactivated the virus within 30 min (*p* < 0.05). Our results therefore showed that all DSP steps for the purification of MV can be performed at room temperature without reducing the stability of the virus.

### 3.3. The pI of MV and the Effect of pH Stress

**Figure 3:**
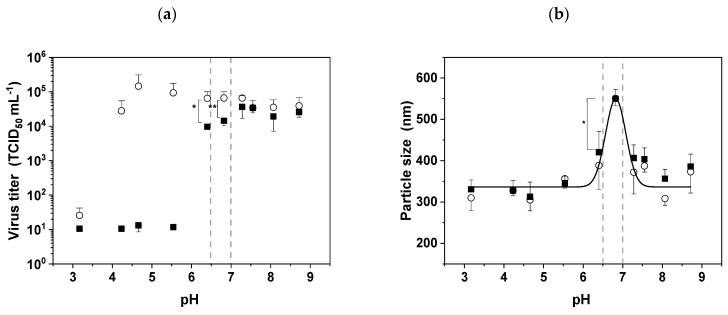
The stability of MV is dependent on pH. (**a**) The infectivity of MV was determined immediately after adjusting the pH (open circles) by diluting MV suspensions 1:10 in the appropriate buffer, and after incubation at room temperature for 1 h (black squares). (**b**) The pH-dependent increase in particle size was measured by dynamic light scattering (DLS) immediately after dilution (open circles) and after incubation at room temperature for 1 h (black squares). Data are means plus standard deviations (*n* = 3; * *p* ≤ 0.05 and ** *p* < 0.1).

The pH is an important DSP parameter, because it can be adjusted to influence the surface charge of MV in the feed stream to facilitate separation based on electrostatic interactions, as long as the virus remains stable. We found that MV was completely inactivated at pH values below 6.0, and that infectivity fell by 0.5 log values within the slightly acidic pH range 6.0–7.0 after 1 h of incubation in contrast to 0 h (pH 6.4: *p* < 0.1 and pH 6.8: *p* = 0.05 ;Figure 3a). In contrast, the virus remained stable for up to 1 h in the basic pH range 7.0–9.0.

It is important to know the pI of MV in order to avoid aggregation and/or to adjust the surface charge during DSP operations involving electrostatic interactions. We calculated the theoretical pI using the corresponding values for the extracellular domains of the two surface proteins: hemagglutinin and fusion protein (Table 1). These proteins assemble on the virus surface in a tetramer/trimer arrangement, suggesting they are present at a ratio of 4:3 [25]. Given this assumption, we calculated that MV has an average pI of 6.7 based on the reported amino acid sequences of the surface proteins [26].

Particle aggregation is most likely to occur if the DSP buffer matches the pI of the virus, so we investigated this phenomenon by DLS (Figure 3b). The typical particle size of 387.2 ± 14.9 nm represents the average size of individual MV particles. Between pH 6.0 and 7.0, the average particle size increased, confirming that aggregation is promoted within this pH range. The maximum particle size was observed at pH 6.8 (550.8 ± 17.1 nm), which is a significant increase (*p* < 0.05) compared to the average size. This empirically determined pI was very close to the theoretical value.

### 3.4. Chemical Stress

#### 3.4.1. Buffer Ionic Strength and Osmolality

**Figure 4:**
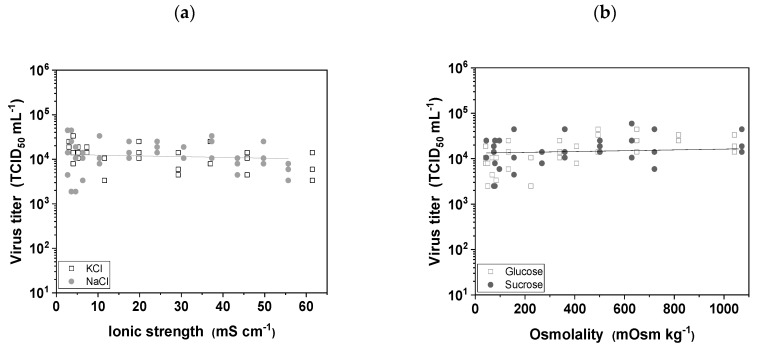
The influence of buffer ionic strength and osmolality on the infectivity of MV. Aliquots of the virus were diluted 1:10 in different salt and sugar solutions for 24 h at 4 °C. (**a**) NaCl and KCl solutions were used to determine the effect of buffer ionic strength. (**b**) Glucose and sucrose solutions were used to determine the effect of buffer osmolality. Experiments were conducted in triplicate, and all data points are shown.

The effects of buffer ionic strength and osmolality were tested to replicate the changing conditions encountered during DSP. We therefore tested the stability of MV in the presence of two salts often used in chromatography elution buffers (NaCl and KCl) and two sugars often used in cell growth medium and/or in formulation buffers (glucose and sucrose). We found that neither variations in ionic strength (Figure 4a) nor variations in osmolality (Figure 4b) influenced the infectivity of MV, and none of the conditions we tested led to a significant reduction in infectivity, which means the virus is robust to the buffer conditions in typical DSP operations.

#### 3.4.2. Buffer Components and Additives

DSP typically requires the use of several different buffers, so we also tested the stability of MV in four common buffers at two different concentrations while maintaining the pH at a constant value of 7.4. The four buffers were Tris, HEPES, phosphate, and citrate-phosphate, and each was tested at 20 and 100 mM. We found that MV remained stable in all four buffers at both concentrations, indicating the virus is broadly compatible with the buffer conditions widely used during DSP (Table 2).

We also tested a range of additives that are commonly used in DSP and/or that enhance the stability of vaccines and other virus preparations (Table 3). We observed a concentration-dependent decline in the infectivity of MV when incubated in the presence of CaCl_2_ and MgSO_4._ The loss of infectivity was strongest in the case of CaCl_2_, resulting in log reduction values of ~0.6 at 0.75 M (*p* < 0.05) and ~3.7 at 1.5 M (*p* < 0.05). The presence of MgSO_4_ resulted in log reduction values of ~0.8 at 0.75 M (*p* < 0.05) and ~1.25 at 1.5 M (*p* < 0.05). NaCl and L-arginine had no effect on the infectivity of MV, even at high concentrations.

### 3.5. Mechanical Stress

DSP involves various forms of mechanical stress, which are imposed by the pumps and tubes that move the feed stream through the unit operations and also by the unit operations themselves as the feed stream passes through various columns and membranes. We therefore investigated the effect of shear stress on the infectivity of MV by separately testing the basic DSP setup (pumps and tubes) and the complete system including a tangential flow filtration (TFF) module.

#### 3.5.1. Shear Stress Induced by Pumping

**Figure 5:**
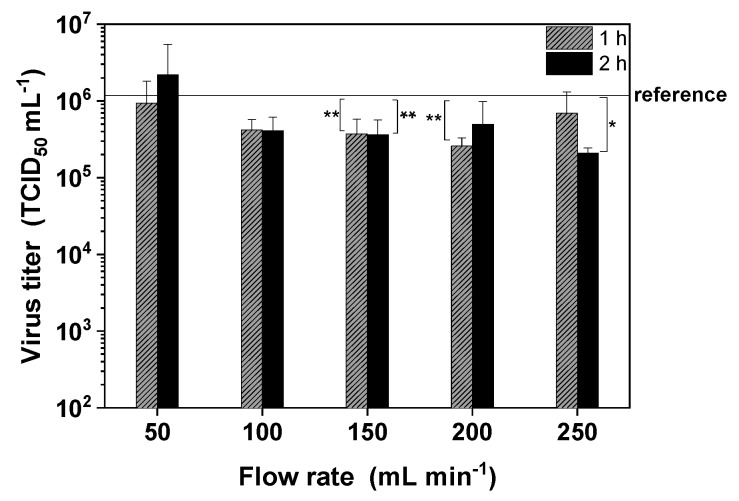
The time-dependent influence of shear stress induced by the peristaltic pump on the infectivity of MV. The virus suspension was pumped in a circle to determine the influence of shear stress caused by the basic downstream processing (DSP) setup. Data are means plus standard deviations of triplicates. The reference is shown as a solid horizontal line (* *p* ≤ 0.05; ** *p* < 0.1).

We tested the effect of a peristaltic pump, because such devices are often used for small-scale filtration. We tested a range of flow rates (50–250 mL min^−1^) and observed only a slight flow-rate-dependent decrease in the infectivity of MV compared to the reference (Figure 5; *p* = 0.05 for 250 mL min^−1^ and 2 h). Only laminar flow conditions were present within the range of flow rates we tested (Re = 172–862 and µ (20 °C) = 1.28 ± 0.03 mPas assuming the density of water at 20 °C is ~998 kg m^−3^). The maximum flow rate of 250 mL min^−1^ generated a shear rate of 383.8 s^−1^ ($R_{id}$ = 2.4 mm), so the maximum average shear stress ($\tau_{av}$) was 491.2 mPa, based on Equations (2) and (3). This is low compared to the typical shear rates in a TFF system (Section 3.5.2), and the main effect may therefore be induced by the peristaltic pump head itself.

#### 3.5.2. Shear Stress in a TFF Module

**Figure 6:**
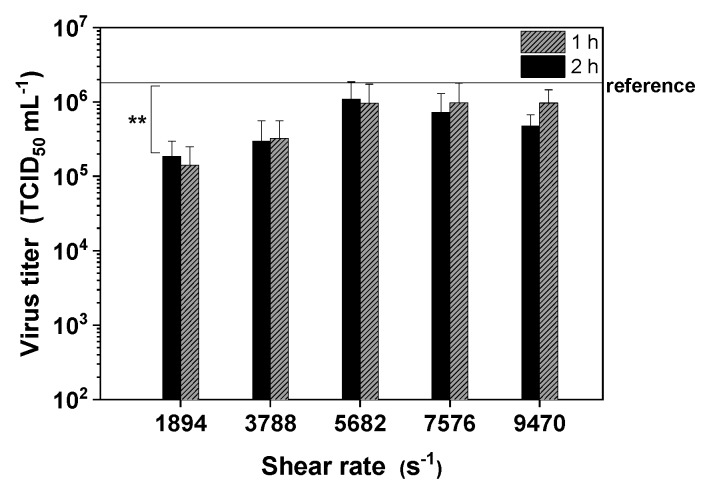
The time-dependent influence of shear rates in a membrane module on the infectivity of MV. The virus suspension was pumped in a circle to determine the influence of shear stress caused by the basic DSP setup with an integrated flat-sheet membrane module. Data are means plus standard deviations of triplicates. The reference is shown as a solid horizontal line (** *p* < 0.1).

Finally, we installed a flat-sheet TFF membrane module to investigate its effect on the infectivity of MV. The aim was to establish an optimal flow rate that was neither too high, risking the inactivation of MV by shear stress, nor too low, risking an increase in adsorptive effects caused by convective transport. MV adsorbed to the membrane at low flow rates (*p* < 0.1 for a shear rate of 1894 s^−1^), but the loss of active MV particles declined as the flow rate increased from 50 to 150 mL min^−1^ (Figure 6). However, increasing the flow rate to 250 mL min^−1^ resulted in the inactivation of the virus. Therefore, a flow rate of 150 mL min^−1^ was optimal for our filtration setup.

Given the assumption that the flow channel within the membrane module is rectangular, we used Equation (4) to determine the effective shear rate [27]. Based on a flow rate of 150 mL min^−1^, a channel width of 200 µm, and a channel thickness of 6.6 cm, the effective shear rate ($\dot{\gamma })$ was estimated as shown in Equation (6), ignoring the influence of the spacer in the membrane module on flow distribution

(6) $$\dot{\gamma }=\frac{6\cdot 2.5\cdot 10^{-6}\;m^{3}\;s^{-1}}{2\cdot 0.033\;m\cdot {\left(200\cdot 10^{-6}m\right)}^{2}}=5681.82\;s^{-1}$$

The average shear stress on MV particles in the rectangular flow channel of flat-sheet membranes can therefore be calculated by including the dynamic viscosity of water, as shown in Equation (7)

(7) $$\tau_{av}=\frac{6\cdot 2.5\cdot 10^{-6}\;m^{3}\;s^{-1}\cdot 1.28\cdot 10^{-3}\frac{N\cdot s}{m^{2}}}{2\cdot 0.033\;m\cdot {\left(200\cdot 10^{-6}m\right)}^{2}}=7.27\;\mathrm{Pa}$$

The shear rates therefore ranged from 1894 s^−1^ (50 mL min^−1^) to 9470 s^−1^ (250 mL min^−1^), and the effective average shear stress within the membrane module ranged from 2.4 Pa (1894 s^−1^) to 12.1 Pa (9470 s^−1^).

## 4. Discussion

### 4.1. Up to Five Freeze–Thaw Cycles Cause no Significant MV Inactivation Even in the Absence of Serum

Freeze–thaw stress is often induced between the upstream and the downstream process or during DSP, and it is important to understand the effect of temperature fluctuations on viruses because protein denaturation occurs at the ice–liquid interface [28]. Rapid freezing in particular is associated with protein loss due to the formation of small ice crystals that increase the ice surface area [29,30]. Furthermore, the crystallization of buffer components changes the pH and osmolality of the medium, and promotes the formation of ice bridges between virus particles, which also affects virus infectivity by damaging surface proteins (required for interactions with target cells) and the fragile lipid envelope [17,31]. We found that MV can withstand up to five freeze–thaw cycles in serum-free medium, and further cycles cause a loss of infectivity. In contrast, MV can withstand at least 10 freeze–thaw cycles in serum-containing medium without any loss of infectivity. This could be due to the known viral protective property of fetal serum components [32]. Similarly, Vaccinia virus was able to withstand four freeze–thaw cycles (thawing for 1 min at 37 °C and freezing for 3 min in dry-ice/ethanol) [33]. However, the Influenza A virus was much more susceptible, with one log reduction in infectivity after two cycles of freezing at –40 °C and thawing for 20 min at room temperature, and a seven log reduction after five cycles [34]. In the case of Vesicular stomatitis virus (VSV), infectivity declined by two log values after 10 freeze–thaw cycles but the precise conditions were not specified [35]. Therefore, it appears that the MV is more robust than the Influenza A virus and VSV with respect to the damage caused by freeze–thaw cycles, which is advantageous for the preparation of the virus particles. Due to the clinical relevance of using a serum-free medium for the preparation of the MV, the fact that MV survives five cycles without a serum is a massive advantage for the preparation of clinical batches of MV for cancer patients.

### 4.2. MV Is Extremely Thermosensitive

Temperature is always an issue in biopharmaceutical manufacturing, particularly in large-scale processes where the unit operations take much longer (hours) compared to processes in the laboratory. Temperature sensitivity is a critical parameter during upstream production because the optimum temperature must balance the sensitivity of the product against the productivity of the cell line [9]. Former MV stability studies therefore concentrated on the typical USP temperatures (up to 37 °C) and the stability of the MV over 5 to 7 days (typical time period for USP). We now focus on the thermal stability of MV within hours and at a broader temperature range (15–65 °C) with regard to the DSP. DSP can be carried out at a uniform lower temperature if necessary, but room temperature unit operations are less expensive. We found that MV remains stable at room temperature for at least 2 h, which is beneficial because this would allow DSP without refrigeration. However, the stability of MV declined at temperatures exceeding 35 °C, and the protective effect of serum observed during freeze–thaw cycles was not apparent. A previous study, focusing on the MV stability in the upstream process [9], confirmed a decline of MV infectivity after incubation at 37 °C for 2 h. Here, the critical temperature for MV was estimated between 35 °C and 45 °C. The infectivity of the particles decreased by 1.5–2 log values when incubated at 45 °C for 2 h regardless of whether or not serum was present in the medium. At 50 and 55 °C, the infectivity of the particles rapidly fell below the detection limit (10^2^ TCID_50_ mL^−1^), representing a log reduction value of at least 4. This probably reflects the denaturation of protein receptors displayed on the virus envelope [19]. Previous studies of MV have also demonstrated extreme thermosensitivity, including tests of MV (Edmonston strain) produced in Hep-2 cells in the presence of 10% fetal calf serum [36], and MV (Sugiyama strain) produced in human amniotic FL cells in the presence of 0.4% bovine serum [37]. Other enveloped viruses are mildly thermosensitive, for example, the infectivity of baculoviruses and Influenza A virus decreased by two log values at 50–56 °C [38,39]. The extreme thermosensitivity of MV is useful knowledge that can be accommodated during DSP by ensuring that all unit operations are conducted at room temperature or below, and that MV suspensions harvested from the bioreactor are cooled and processed without delay or stored frozen batch-wise for further processing. Based on this FD study, we conducted several filtration and chromatography unit operations at room temperature and confirmed that the MV remained stable at this temperature also under real DSP conditions.

### 4.3. The pI of MV Is ~6.8 and the Virus Is Highly Sensitive to Acidic Media

The pH sensitivity of MV defines the DSP working pH range. We found that MV started to lose infectivity as soon as the buffer pH fell below 7.0, and complete inactivation was observed at pH 6.0, supporting the results of earlier studies [9,18]. These studies investigated the pH dependency on MV infectivity but not the pH stability of MV together with its aggregation behavior as we did. This is important knowledge in the context of DSP, because it will facilitate the development of charge-dependent process steps such as ion exchange chromatography while ensuring that acidic buffers are avoided. The sensitivity of MV in the presence of acidic buffers may again reflect the denaturation of surface receptors under such conditions. Other enveloped viruses are also sensitive to acidity. For example, baculoviruses are most stable at pH 7.0, and infectivity is reduced by two log values at pH 5.0 [40], whereas Influenza A virus loses infectivity at pH < 6.0 [39]. Other enveloped viruses are robust against extreme pH, including Lumpy skin disease virus, which maintains infectivity in the pH range 4.0–8.0 and remains stable in the pH range 2.0 to 10.0 for 14 days at 0–4 °C [41].

The pH of DSP buffers must also be adjusted to avoid virus aggregation, which is promoted if the buffer pH matches the pI of the virus and its overall surface charge is therefore zero. It is advantageous to know the pI of the virus in order to develop appropriate DSP steps, and accordingly we predicted a value of 6.7–6.8 based on the average pI of the virus surface proteins adjusted according to their relative abundance. A value of ~7 has previously been reported in a technical note, based on isoelectric titration [42]. Interestingly, we did not observe the strong inactivation of MV when the buffer was adjusted to match the pI, although DLS experiments revealed a peak of particle aggregation. This contrasts with the results reported for baculoviruses, where aggregation coincided with virus inactivation [40]. However, MV and baculoviruses differ not only in terms of morphology but also in the position of the surface receptors required for infectivity. MV is spherical and is uniformly covered with receptors, whereas baculovirus is rod-shaped and the receptors are clustered on one side. The even distribution of surface receptors allows MV to remain infective even when present as aggregates. The pI of most enveloped viruses is ≤6.0, for example, *Autographa californica* nucleopolyhedrovirus (a baculovirus) has a pI of 5.4, Moloney murine leukemia virus (a retrovirus) has a pI of ~6, and the modified vaccinia Ankara virus (poxvirus) has a pI in the range 2.3–5.0 depending on the antigen it displays [43]. The pI of MV is at the higher end of the typical range for enveloped viruses (3.5–7) and is similar to that of the H3N1 strain of Influenza A virus, which ranges from pH 6.5 to 6.8 [43,44]. The relatively high pI of MV together with its sensitivity to acidic buffers means it is not possible to confer an overall positive charge on the MV particle during DSP. Above its pI, MV is negatively charged and remains infectious until the buffer exceeds pH 9.0, which suggests that anion-exchange chromatography is suitable as a DSP step. Even so, the overall negative charge distribution on MV can be reduced at pH values slightly above the pI because the extracellular domain of the fusion protein has a theoretical pI of 7.0–7.4 [26]. This may also enable the binding of MV particles to cation-exchange media in the presence of weak counter-ions. We confirmed this, as we were able to bind MV to cation-exchange matrices by shifting the pH of the suspension near the pI. In other unit operations, which are not dependent on charge, maintaining the buffer pH above 6.8 should avoid particle aggregation.

### 4.4. MV Tolerates a Broad Range of Buffer Types, Osmolality and Ionic Strength

Buffer osmolality and ionic strength are interrelated factors that can affect the infectivity of viruses, for example, by promoting/inhibiting interactions with charged surfaces and by promoting water flux into or out of the virus particle [19,40,45]. We found that the osmolality and ionic strength of the buffer had no impact on MV infectivity, indicating that low-salt and high-salt buffers for chromatography or diafiltration are compatible with MV and will not affect its infectivity, at least at 4 °C and for 24 h. We also found that MV was stable in four different buffers, indicating that the buffer type can be chosen freely for different DSP operations [46]. It may be necessary to evaluate the buffers again for long-term storage. In contrast, we found that the type of salt used in the buffer must be selected carefully. We found that high concentrations of MgSO_4_ and CaCl_2_ caused the inactivation of MV. The negative effect of CaCl_2_ has been reported before [47], but earlier studies suggested that 1 M MgSO_4_ has a protective effect on MV at 50 °C [47], that even high concentrations have no adverse effect [9], or that 1 M MgSO_4_ has at most a minimal negative effect at room temperature [48]. Therefore MgSO_4_ appears to protect MV from heat inactivation but carries an inherent ability to inactivate the virus. NaCl is typically used as an eluent in ion exchange chromatography or as a high-salt diafiltration buffer, and we found that not even high concentrations (up to 2 M) affected the infectivity of MV, in agreement with previous experiments [9].

We also tested L-arginine, which is used as a stabilizer in vaccine formulations and is known to prevent the aggregation of proteins [49,50,51]. L-arginine at concentrations of up to 0.2 M did not cause the inactivation of MV and can therefore be used for MV purification and/or formulation to prevent particle aggregation. However, 1 M L-arginine was previously shown to reduce the infectivity of MV (Edmonston-Zagreb strain) produced in Vero cells [48].

### 4.5. Shear Stress in the Membrane Module Has a Significant Impact on Recovery Infectious MV Infectivity

Mechanical stress during DSP reflects a combination of shear stress caused by pumps that move the feed stream between different unit operations and shear stress that occurs within units such as filter modules, where the liquid is forced through narrow channels. It is important to investigate the effect of such factors on product quality, and indeed this is recommended by ICH guidelines [21].

We began by testing the effect of shear stress caused by the standard process equipment. Peristaltic pumps are often used in small-scale purification processes to introduce the feed stream into filter modules and chromatography columns/cassettes. We found that pumping the MV suspension around the DSP system had only a minor impact on infectivity, suggesting that the mechanical stress was insufficient to severely disrupt the virus surface receptors and lipid envelope. The potential impact of pump-related shear stress on MV has been raised in one previous study [18], but we are unaware of any reports describing the testing of this factor in isolation. However, another study using the same peristaltic pump reported no significant effects of shear stress on baculoviruses [38]. We calculated a maximum pump-related shear stress of ~0.49 N/m^2^ in the tubes, which is very low compared to computational fluid dynamics (CFD) models of other pumps (e.g., a shear stress range of 50–1000 N/m² was estimated by CFD modeling for a roller pump [52]). This supports the hypothesis that shear stress induced by peristaltic pumps is not primarily associated with the flow but with the pump head, which causes shear stress peaks that may reduce MV infectivity.

TFF with PES membranes is widely used as virus purification method, but the PES substrate tends to adsorb proteins (including those on the surface of enveloped viruses) by non-specific binding, even though PES has a low non-specific binding capacity compared to other materials. Accordingly, we found that the PES membrane adsorbed MV at a low flow rate of 50 mL min^−1^, but adsorption was inhibited at higher flow rates. However, increasing the flow rate too much also increased the shear stress, to a maximum of 12.1 N/m^2^ in the membrane module at a flow rate of 250 mL min^−1^. We therefore found that a flow rate of 150 mL min^−1^ was optimal, eliminating non-specific adsorption without significantly reducing the infectivity of the recovered virus. Similar optimal TFF flow rates were also reported for Influenza A virus [27] and baculoviruses [53]. Purification studies carried out in our lab using this shear rate/flow rate showed good recovery of MV.

## 5. Conclusions

We have shown that FD studies are useful for the establishment of optimal DSP conditions for the purification of enveloped viruses, specifically MV. FD studies are a starting point to develop a purification strategy and build the basis of an efficient downstream process. The purification of MV is challenging, because many factors contribute towards the inactivation of the virus. The treatment of cancer using oncolytic MV requires a large number of infectious particles, so it is necessary to understand the critical process parameters during the early stages of process development. We found that several process parameters were not critical (five freeze–thaw cycles, the type of buffer, and the ionic strength and osmolality of salt (NaCl/KCl) and sugar), which offers a degree of flexibility during process development. However, we also identified critical process parameters that significantly affected the infectivity of MV: temperature, pH, the presence of certain salts (CaCl_2_/MgSO_4_), and shear stress. We found that MV was rapidly and completely inactivated at temperatures exceeding 40 °C, even in the presence of serum, indicating that the purification of MV from the culture medium should begin with a cooling step immediately after harvesting. However, we saw no evidence of inactivation at temperatures below 35 °C in incubations lasting up to 2 h, suggesting that all DSP steps can be carried out at room temperature within this time frame. It is therefore unnecessary to cool the entire DSP to 4 °C. Importantly, our results indicated a narrow DSP working pH range of 7.0–9.0 for MV, reflecting its relatively high pI of ~6.8, ruling out several unit operations that require acidic buffers. We also found that MV is shear sensitive, indicating that each DSP unit operation should be evaluated carefully to determine the conditions that reduce shear stress. For the purification of MV by TFF, the optimal flow rate was 150 mL min^−1^—this prevented the non-specific adsorption of the virus but limited the shear rate in the membrane module to ~5700 s^−1^. The identification of these critical parameters will facilitate the development of an effective DSP strategy for MV and supports the use of FD studies as an integral part of early-stage process development for each virus candidate. This approach will ensure product quality and reduce losses during product purification.

## Figures and Tables

**Table 1:** MV has two surface proteins: hemagglutinin and the fusion protein. The theoretical pI was calculated for the extracellular domains of these two proteins based on the amino acid sequences [26].

Protein	Domain	Length (Amino Acids)	Theoretical (Average) pI
Fusion (P69353.1)	Extracellular	471	7.4
Hemagglutinin (P08362.1)	Extracellular	559	6.2

**Table 2:** Influence of different buffers (pH 7.4) at two different concentrations on the infectivity of MV. Data are means plus standard deviations of triplicate.

Buffer	Concentration (mM)	Log_10_ TCID_50_ mL^−1^
PBS (reference)		5.69 ± 0.19
Tris	20	5.44 ± 0.07
	100	5.69 ± 0.26
HEPES	20	5.73 ± 0.26
	100	5.65 ± 0.13
Phosphate buffer	20	5.61 ± 0.19
	100	5.44 ± 0.14
Citrate-phosphate buffer	20	5.48 ± 0.07
	100	5.48 ± 0.14

**Table 3:** Influence of different additives on the infectivity of MV. Data are means plus standard deviations of triplicates(* *p* < 0.05).

Additive	Concentration (M)	Log_10_ TCID_50_ mL^−1^
NaCl	0.5	5.4 ± 0.1
	1	5.5 ± 0.1
	2	5.8 ± 0.2
CaCl_2_	0.375	5.7 ± 0.3
	0.75	5.0 ± 0.1 *
	1.5	2.0 ± 0.1 *
MgSO_4_	0.375	5.8 ± 0.4
	0.75	5.0 ± 0.3 *
	1.5	4.6 ± 0.1 *
L-Arginine	0.1	5.6 ± 0.1
	0.2	5.4 ± 0.1
